# The Evolving Concept of Neuro-Thromboinflammation for Neurodegenerative Disorders and Neurotrauma: A Rationale for PAR1-Targeting Therapies

**DOI:** 10.3390/biom11111558

**Published:** 2021-10-21

**Authors:** Barry W. Festoff, Chris Dockendorff

**Affiliations:** 1 PHLOGISTIX LLC, Department of Neurology, University of Kansas Medical School, Kansas City, MO 64108, USA; 2 Function Therapeutics LLC, Milwaukee, WI 53202, USA; cdockendorff@function-therapeutics.com

**Keywords:** neurodegeneration, neurotrauma, ALS, inflammation, thromboinflammation, neuro-thromboinflammation, PAR1, APC, 3K3A-APC, parmodulin

## Abstract

Interest in the role of coagulation and fibrinolysis in the nervous system was active in several laboratories dating back before cloning of the functional thrombin receptor in 1991. As one of those, our attention was initially on thrombin and plasminogen activators in synapse formation and elimination in the neuromuscular system, with orientation towards diseases such as amyotrophic lateral sclerosis (ALS) and how clotting and fibrinolytic pathways fit into its pathogenesis. This perspective is on neuro-thromboinflammation, emphasizing this emerging concept from studies and reports over more than three decades. It underscores how it may lead to novel therapeutic approaches to treat the ravages of neurotrauma and neurodegenerative diseases, with a focus on PAR1, ALS, and parmodulins.

## 1. Introduction to Neuro-Thromboinflammation and Historical Background

Our initial concept began with visualizing synapse formation, such as the formation of blood clots, which involved thrombin-mediated coagulation. Likewise, in the context of synapse retraction or “dying back” as a mechanism for amyotrophic lateral sclerosis (ALS) pathogenesis, synapse elimination was viewed in a similar way to clot retraction [1]. We predicted that one or more molecular members of the clotting cascade pathways would be found at the synapses and turned to the classical model, the rodent neuromuscular junction (NMJ) model. The hypothesis also predicted that as with clot retraction, synapse retraction, or elimination, would likely involve components of fibrinolytic pathways. Those early studies led to several discoveries: plasminogen activators (PAs), primarily urokinase (uPA), were developmentally regulated at neonatal mouse NMJs [2]; the principal tissue inhibitor of thrombin, protease nexin I (PNI; serine protease inhibitor E2—serpinE2) was also developmentally localized at NMJs [3]; thrombin activity correlated with postnatal-activity-dependent synapse elimination in a Hebbian manner at the NMJ [4,5]. The thrombin- and PA-inhibiting serpin, PNI, was also developmentally regulated and inversely correlated with synapse elimination [6]. These findings were of interest to the phenomena associated with synapse elimination, as reviewed in [7]. From the perspective of the thrombin–PNI balance, and where thrombin might be gaining access to the NMJ synaptic cleft, we subsequently found that prothrombin was expressed by developing skeletal muscle [8]. Other groups also began pursuing these findings as well [9,10]. We found that this same serpin, PNI, also rescued avian motor neurons from naturally occurring and axotomy-induced cell death [11]. It was also stimulated to be secreted from astrocytes in the CNS, where it was an abundant protein, by vasoactive intestinal polypeptide (VIP) [12]. Contemporaneous with our findings, others had found that a similar serpin was expressed in mouse seminal vesicle [13], raising the possibility of a “duplicate“ gene. We then showed that both the mouse brain and seminal vesicle genes were the same [14].

Our results, along with those of the laboratories of Dennis Cunningham at UC, Irvine, and Denis Monard at the Friederich Miescher Institute in Basel, amongst others, culminated in the first symposium bringing together basic and clinical neuroscientists with coagulation and fibrinolysis experts under the aegis of NATO [15]. That meeting, which took place in Maratea, Italy, in 1989, allowed for the sharing of ideas between very disparate fields and expertise. Most neuroscientists and clinicians thought that the terms thrombosis, hemostasis, and coagulation were interchangeable but learned otherwise. As Fisher has written [16], thrombosis involves clot formation within a blood vessel; hemostasis is avoidance or arrest of bleeding within a blood vessel; coagulation indicates a liquid conversion to a gel, semi-solid, or solid mass. With our discovery that thrombomodulin (TM) was functionally active on astrocyte cell surfaces, we proposed a microcoagulation cascade within the CNS [17] that ultimately became clarified with the discovery of the “thrombin receptor” on cells [18,19]. This G-protein-coupled receptor (GPCR), now known as protease-activated receptor 1 (PAR1), is activated by cleavage of its N-terminus by proteases such as thrombin, revealing a “tethered ligand” peptide [20,21]. We showed that thrombin activates PAR1 on astrocytes, causing shape changes (reversal of stellation) [22], and that this effect is mediated by protein kinase C β-1 [23].

Perhaps inspired in part by the Maratea meeting, much progress has been made related to coagulation-related enzymes, receptors, and inhibitors in the nervous system, with additional groups making important contributions. This has culminated in recent designations for the “coag-inflamm nexus” [24], with terms such as “coag-neurology”, the “neuro-glial coagulonome”, and neuro-thromboinflammation used to describe this emerging field. These all point to the critical roles that coagulation and inflammation play in neural development and the pathogenesis of neurologic diseases. Central to this is the evidence that PARs represent a critical link between these two systems, coagulation, and inflammation. The continued elucidation of their molecular mechanisms will be essential in furthering knowledge of the growing field of neuro-thromboinflammation. Importantly, after almost 40 years, we may be seeing the application of these discoveries to the treatment of one or more neurologic disorders. This article and others in this issue are a testament to the novel work of those endeavoring to understand this field and to translate findings to the clinic.

### 1.1. The Interaction between Inflammation and Coagulation Is a Continuum

Beyond exhibiting a significant degree of coordination in signaling and regulatory pathways following both tissue injury and pathogen invasion, once activated it may be difficult to turn off excessive inflammation and coagulation when these two systems interact as a continuum. In severe disorders such as sepsis and sterile systemic inflammatory response syndrome (SIRS), as occurs with trauma, inflammation (usually driven by a damaged endothelium or activated immune cells) activates coagulation, downregulates endogenous anticoagulants, and inhibits fibrinolysis. These are the three main mechanisms by which inflammation influences clotting. In turn, α-thrombin, the final serine protease in the clotting cascade, activates specific cell surface GPCRs, the PARs, and is a potent proinflammatory mediator [25,26].

### 1.2. The Coevolution of Coagulation, Innate Immunity, and Inflammation

A major evolutionary advantage that protects all mammals from invading pathogens uses two critical host defense systems: innate immunity or inflammation and coagulation. These systems are linked throughout evolution and one of the best examples of this is found in the ancient horseshoe crab (*Limulus polyphemus*) that is so abundant on the mid-Atlantic to New England shorelines in North America. The bedrock upon which the theory that coagulation and innate immune inflammatory responses form a nexus is found in this arthropod horseshoe crab, which has existed for 450 million years. Similarly to other invertebrates, the horseshoe crab lacks an adaptive immune system and its host defense is wholly provided by its innate immunity [27].

Unlike vertebrates that possess an open immune or lymphatic system and a closed circulatory one, Limulus has a linked semi-closed system that is open to sea water in the aquatic world in which it lives. This environment is teeming with bacteria as well as highly inflammatory lipopolysaccharides (LPSs), commonly referred to as endotoxins. Limulus protects itself via a highly sensitive means to detect LPS, based in a single cell in its blue hemolymph blood, the amebocyte. This cell acts similarly to macrophages and platelets and contains clotting factors that are released when it detects and encounters endotoxins. This led to discoveries at the Marine Biology Laboratory (MBL) at Woods Hole, MA, in the 1950s and 1960s by Frederik Bang and Jack Levin of Johns Hopkins University. Ultimately, this produced the Limulus Amoebocyte Lysate (LAL) assay or limulus lysate test, which is still in use worldwide for the detection of femtomolar concentrations of endotoxins [28,29].

### 1.3. Clinical Identification and Application of the Coag-Inflamm Nexus

**Figure 1:**
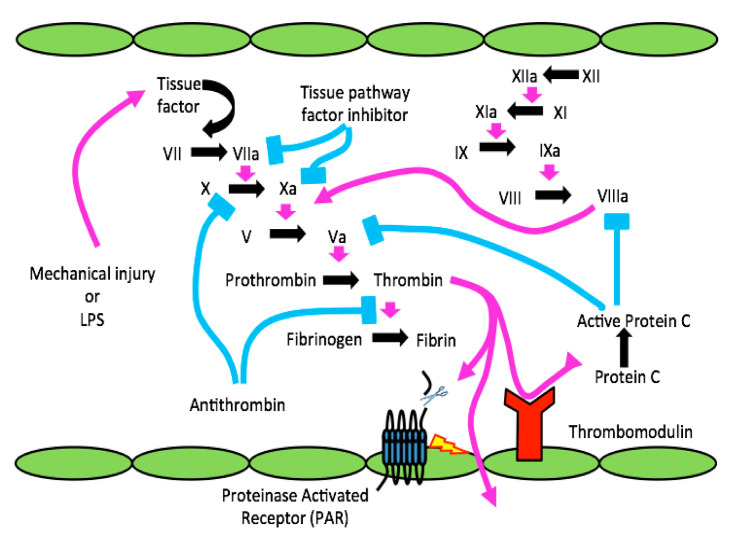
The clotting cascade activated by injury or infection (e.g., by LPS) and the blood–brain barrier (BBB). Schematic showing infection, LPS, or injury-releasing tissue factor (TF) to activate clotting, resulting in active α-thrombin, which cleaves PAR1 in endothelial cells (ECs) to disrupt the BBB. Through disrupted BBB, α-thrombin gains access to the CNS, where it can cleavage-activate PARs on microglia or astrocytes, promoting neuroinflammation and potentially forming neurodegenerative lesions (modified from Festoff and Citron, 2019 [24]).

While Limulus provides the evolutionary confirmation, the coag-inflamm nexus is recognized in human clinical medicine, with evidence that inflammatory mediators generate procoagulant signals, while intravascular thrombosis activates multiple components of the innate immune system [28,29,30]. This nexus is integral to the dangerous thromboinflammation associated with the current COVID-19 pandemic [31,32,33]. A closely related example is the global bleeding initiated by severe infection observed in sepsis, with concomitant disseminated intravascular coagulation (DIC), the hallmark of which is diffuse microthrombi with simultaneous consumption of coagulation components [34]. DIC can be treated by anticoagulants, such as heparin, or a recombinant form of thrombomodulin (rTM, ART-123, Recomodulin^®^) approved to treat DIC in Japan. Figure 1 schematically shows the intrinsic and extrinsic intravascular coagulation pathways activated either by sepsis (LPS) or injury; however, anticoagulant (or more broadly, antithrombotic) approaches are presumably always limited by their increased risk of bleeding, as in the case of the anticoagulant-activated protein C (APC), which was withdrawn for the treatment of sepsis [35]. Alternatively, anti-inflammatory approaches to treat sepsis or COVID-19 and prevent DIC may be promising but have thus far seen only limited successes; dexamethasone and the IL-6 inhibitor tocilizumab have shown no or only modest improvements in mortality for patients with severe COVID-19 as of June 2021 [36,37]. Therapies with the potential to safely modulate both thrombosis and inflammation are highly desirable within the context of sepsis, COVID-19, and other diseases.

## 2. Key Players in Neuro-Thromboinflammation

### 2.1. Tissue Factor (TF)

Tissue factor (TF), also called clotting factor III, CD142, or tissue thromboplastin (TTP), is a 45 kilodalton transmembrane glycoprotein that serves as a key activator of the coagulation cascade [38,39]. It is present on the surfaces of several cell types, in particular white blood cells (WBCs), subendothelial fibroblasts, endothelial cells (ECs), and also on microparticles released by activated or apoptotic cells. TF becomes the primary initiator of the extrinsic blood clotting cascade by binding to and activating factor VII, then the resulting complex activates factor X, which then activates factor V to activate prothrombin, as shown in Figure 1. With microvascular damage or activation from bacterial invasion or injury, exposed TF from subendothelial tissues or expressed on activated ECs largely accounts for coagulation activation by the extrinsic clotting system. The TF–FVIIa complex can also proteolytically cleave PARs, which is thought to occur via a complex composed of the endothelial protein C receptor (EPCR), TF, FVIIa, and FXa [40].

While TF is now known as the initiator of inflammation-induced thrombin generation, it was not always considered so, since bacteria or LPS (endotoxin) was deemed the culprit. TF expression on ECs is induced by contact with cytokines and circulating factors such as C-reactive protein (CRP), tumor necrosis factor alpha (TNF-α), and advanced glycosylated end products (AGEs) [39]. Of relevance to this review, TF is highly enriched in the brain and is important in the concept of neuro-thromboinflammation. In the brain, TF is expressed by astrocytes [41], although it is unclear whether resting or reactive astrocytes are more involved in TF production.

### 2.2. Pathogen-Associated Molecular Patterns (PAMPs)

Critical in the clinical link between coagulation and the innate immune system is that the latter recognizes highly conserved structures known as pathogen-associated molecular patterns (PAMPs) [42]. PAMPs, as postulated by Charles Janeway in 1989 [43], were “signal 0” for innate immune cells, which then led to later “signals” to activate adaptive immunity. PAMPs include the prototypic LPS, as mentioned above, as well as bacterial DNA, viral double-stranded RNA, and peptidoglycan. They bind to cellular pattern recognition receptors (PRRs) such as Toll-like receptors (TLRs), NOD-like receptors, RIG-I-like receptors, AIM2-like receptors, and the receptor for advanced glycation end products (RAGE) on or in innate immune cells. One of the primary functions of innate immunity is to recognize PAMPs through PRRs and to activate rapid defensive mechanisms (recruiting immune cells and activation of the complement cascade), but also to activate adaptive immunity. The innate immune response is essentially antibody-independent and identifies non-self via PAMPS presented by bacteria, or as we will see later, damage-associated molecular patterns (DAMPS) displayed by dying or necrotic cells.

### 2.3. DAMPS, Key in Acute and Chronic Neuro-Thromboinflammation

**Figure 2:**
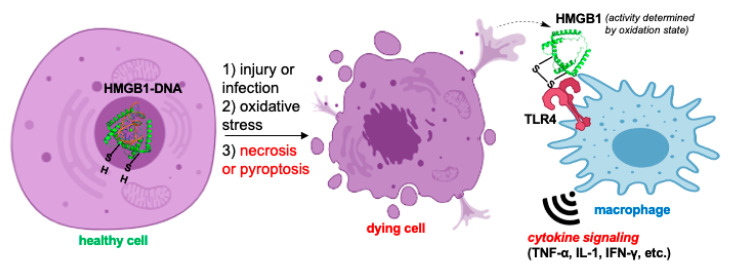
Inflammatory signaling driven by HMGB1. A damaged cell can undergo oxidative stress, resulting in oxidation of HMGB1 to its various disulfide forms, release from DNA and chromatin, and passive diffusion from a dying cell. It is subsequently recognized by TLRs and RAGE in immune cells and other cell types, initiating inflammatory signaling.

**Figure 3:**
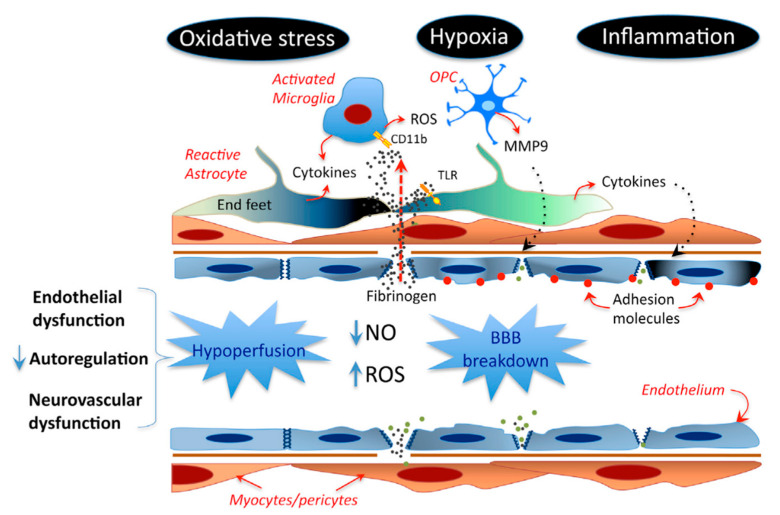
Schema of the BBB/NVU. ECs are shown in blue (lining blood vessels). Inflammatory conditions triggered by hypoxia can compromise tight junctions and create openings in the BBB. Subsequent entry into the brain of certain compounds such as fibrinogen can activate cells (including microglia and astrocytes) to generate cytokines, amplifying the inflammatory response and potentially further compromising the BBB Toll-like receptors (TLRs), matrix metalloproteinase 9 (MMP9), microglia markers (e.g., CD11b), nitric oxide (NO), and reactive oxygen species (ROS).

Just as PAMPs interact with PRRs in infectious states and sepsis, DAMPs engage the innate immune system signaling pathways in sterile inflammation, such as systemic inflammatory response syndrome (SIRS) following poly-trauma or traumatic brain injury (TBI) [44]. The resolution of SIRS acutely begins with the “signal 0” molecule, such as high-mobility group B protein 1 (HMGB1) (Figure 2), a prototypic DAMP that is passively released from necrotic or damaged cells and actively from immune actors such as dendritic cells [45]. HMGB1 activates PRRs and triggers important intracellular signaling pathways (signals 1–4), such as might be the case in simple, single mild TBI that completely resolves going from signal 0 through signals 1–4, ending in resolution with signal 5. Alternatively, it might be more prolonged and aggravated in the evolution to neurodegenerative diseases such as Alzheimer’s disease (AD) and amyotrophic lateral sclerosis (ALS). This is schematically shown at the BBB in Figure 3. HMGB1, a member of the extended HMG-box-containing proteins, was first described as a chromatin-associated molecule, and is translocated from the nucleus to the cytoplasm and then released as described above [46]. HMGB1 is highly conserved and shares 99–100% identity between mice, rats, and humans. When released into the extracellular space, its actions depend on its redox state [47], which determines its binding partners, including receptors such as TLRs 2/4 and 9 and RAGE. In fact, its oxidative state determines its inflammatory roles, since when reduced it acts as a chemokine and can partner with CXCL12 to attract leukocytes (WBCs), or if it is partially oxidized it functions as a cytokine and forms disulfide bonds after binding to TLR4 [48]. These varied functions are critical in the CNS and are illustrated in part in Figure 2.

HMGB1 is also translocated to mitochondria, where it is involved in “quality control” and in autophagy or mitophagy. These are viewed as survival mechanisms, which are also associated with cell death, specifically “autosis”. This can be indicated by the staining of cytoplasmic autophagosomes in dying cells [49]. This is important in neurodegeneration, since frontotemporal dementia (FTD) and ALS-associated C9orf72 hexanucleotide expansion is implicated in autophagy, as will be detailed below [50].

Relevant to ALS, HMGB1 levels were found to be elevated in spinal cord tissue of SOD1-G93A transgenic mice, as well as other proteins associated with HMGB1 pathways such as TLR2/4 and serine/threonine kinase 30, the latter being an ortholog of RAGE [51]. It has also been reported that HMGB1 is elevated in both rat [52] and mouse models of spinal cord injury (SCI) [53], which is not surprising given the extent of neuro-thromboinflammation associated with SCI. In addition to injury-induced markers such as HMGB1, humans may produce autoantibodies (autoAbs) to DAMPs such as HMGB1. This was found to be the case with ALS, whereby patients with elevated serum levels of such autoAbs may correlate with slower progression of the disease [54].

### 2.4. Thrombomodulin (TM), Activated Protein C (APC), and Endothelial Cell PC Receptor (EPCR)

Thrombomodulin (TM), a large transmembrane chondroitin sulfate proteoglycan (CSPG) with five distinct domains, is a key molecule in the regulation of the coag-inflammatory nexus. Originally discovered on luminal EC surfaces, it is abundant in a number of cells from placental trophoblast to keratinocytes and megakaryocytes and platelets [55,56,57]. We found its novel expression and functional activity on brain astrocytes [17], where it could serve as a marker for injury-induced reactive astrocytosis [58]. As with many surface molecules, TM can be released into the circulation as a soluble variant(s) (sTM), largely due to activity of leukocyte elastase or cathepsin G, but also matrix metalloproteases (MMPs) and intramembranous serine proteases known as rhomboids. sTM therefore acts as a marker of endothelial damage [59,60,61,62], as seen in peripheral artery disease [63]. Sernau et al. concluded that sTM is a marker of microvascular, not macrovascular, EC damage [64].

Referring again to Figure 1, the homeostatic balance of coagulation (and inflammation, as we will see) is naturally provided by the anticoagulant serine protease activated protein C (APC) [65,66]. To act as a brake on its procoagulant activity, thrombin cleaves and activates protein C (PC). This is thought to occur at the luminal surface of endothelial cells, with the interaction of thrombin with TM, and of PC with endothelial protein C receptor (EPCR). [56,57]; that is, when thrombin, a procoagulant protease, binds to TM, the resulting complex is no longer procoagulant but alternatively it initiates anticoagulant processes by activating PC, which is bound to adjacent EPCR, to APC. Also shown on EC surfaces is one of several PARs, in this case PAR1, a GPCR for activated α-thrombin [67], as discussed below. From the perspective of blood clotting, activated PC (APC) plays a major role in its regulation, since it inactivates factors Va and VIIIa, while simultaneously promoting fibrinolysis by inactivating the serpin plasminogen activator inhibitor 1 (PAI-1). The take-home message from these results is that APC inhibits coagulation and activates the fibrinolytic pathway. As we discuss in more detail below, it is also cyto- and neuroprotective by cleaving PAR1 at a different site than thrombin does.

### 2.5. Intact and Damaged Microvascular Endothelium

The intact microvascular endothelium, including ECs of the blood–brain barrier (BBB), has an antiadhesive phenotype that uses several mechanisms to neutralize coagulation: (i) thrombin’s principal inhibitor (serpin) in blood, antithrombin (AT), binds to glycosaminoglycans (GAGs) such as heparin sulfate on EC surfaces to inactivate α-thrombin and block activation of FX to FXa, as well as other coagulation proteases (Figure 1); (ii) α-thrombin is converted from a procoagulant to anticoagulant protease by binding TM and the EPCR; (iii) APC is a potent anticoagulant that proteolytically inactivates FVa and FV; (iv) occupancy of EPCR by APC induces cytoprotective signaling through PAR1 to increase endothelial barrier function, thereby preventing increased permeability or “leakiness.” Furthermore, the normal, healthy endothelium expresses high levels of tissue factor pathway inhibitor (TFPI), a multivalent, Kunitz-type serine protease inhibitor that inhibits formation of the TF/FVIIa complex and the activation of prothrombin by prothrombinase, thereby preventing coagulation initiation and subsequent α-thrombin generation.

When the endothelium becomes dysfunctional, a number of markers become elevated in the plasma. One of the most significant is TM, and various sized fragments of sTM circulate in the bloodstream and can be assayed by various means. We found that patients with relapsing, remitting multiple sclerosis (RMMS) release large amounts of sTM, more than patients with systemic lupus erythematosus (SLE) [68], and the highest levels were found in RMMS patients in remission taking glatiramer acetate.

Accordingly, evidence indicates that ECs are pivotal in the coagulation response in systemic inflammation, whether infective in origin (sepsis) or sterile, such as in post-trauma SIRS. The innate immune response in general and thromboinflammation specifically represent critical challenges both for regenerative medicine as well as the use of biomaterials. The simple reason is that both the intrinsic (contact) and extrinsic (TF) pathways are activated. Heparin, a sulfated glycosaminoglycan (GAG), has been used for many decades; however, resistance to heparin has surfaced, especially in the SARS-CoV-2 COVID-19 pandemic, where thromboinflammation is robust, creating a need for alternative strategies [69]. These connections between infection and injury may also be relevant to neuro-thromboinflammation. Organ injuries in the lungs, gut, kidneys, and even the brain indicate that microvascular EC injury may play a key role in pathogenic mechanisms of these disparate tissues.

### 2.6. Protease-Activated Receptors (PARs)

**Figure 4:**
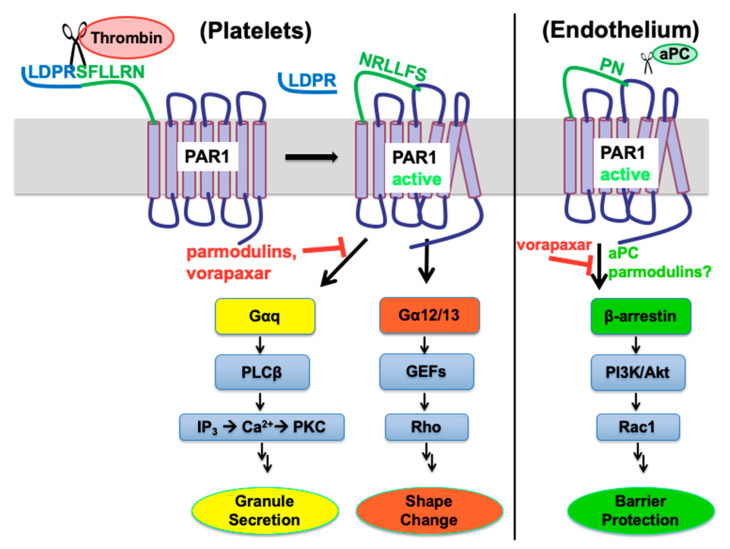
Abbreviated PAR1 signaling diagram. In platelets (**left**), cleavage of the N-terminus of PAR1 by thrombin at Arg41 leads to platelet shape change and granule secretion. These processes can be blocked by PAR1 antagonists such as vorapaxar and by parmodulins to varying degrees. In endothelial cells (**right**), activated protein C (aPC) can cleave PAR1 at Arg46, giving a barrier-protective signal via β-arrestin-2.

PARs, of which there are four subtypes, are GPCRs present in the membranes of numerous cell types, most notably platelets, endothelial cells, and immune cells, but also in all CNS cells [70]. Rather than activation via exogenous ligand binding, activation of PARs occurs via protease-mediated cleavage of the N-terminus of the receptor [18]. Depending on the protease, this exposes one of several possible tethered peptides that act as endogenous ligands and activate a variety of signaling cascades, mediated by G proteins and β-arrestins [71]. Thrombin-driven cleavage and activation of PAR1 leads to granule secretion and platelet shape change (Figure 4, left). This results in rapid clot formation and amplification of the coagulation signal. For these reasons, PAR1 antagonists have been studied for use as antithrombotic agents [72,73,74,75,76], and in 2014 vorapaxar (Zontivity™) was approved by the FDA for coronary artery disease [77,78,79]. PAR2 has been a target for anti-inflammatory compounds [80], and more recently PAR4 on platelets has been successfully targeted by clinical antithrombotic agents [81]; however, this perspective focuses on the connection of PAR1 to neuro-thromboinflammation.

### 2.7. Biased PAR1 Signaling

One of the most important findings in GPCR pharmacology that has emerged is that these receptors can activate a range of intracellular G protein and β-arrestin transducers, while each agonist–receptor pair has a particular bias for the activation of different transducers. This bias can be further affected by the local environment of the receptor via allosteric effects. In this manner, GPCR ligands can be identified with a particular signaling bias that could maximize a desired signaling pathway while minimizing pathological signals. In cultured human ECs, thrombin activation of PAR1 stimulates coupling to Gα12/13 along with Gαq, with downstream activation of the RhoA small G protein. This is the mechanism for thrombin’s increasing vascular permeability and transient disruption of endothelial barriers such as at the BBB [82,83], which occurs at both tight and adherens junctions, as well as reorganization of the actin cytoskeleton. Rezaie and colleagues initially found that whichever ligand is bound to EPCR, it switched the signaling specificity of thrombin from vascular-permeating to barrier-protecting [84], which led to their further discovery that when EPCR is bound by PC, PAR1-cleavage-dependent protective signaling could be mediated by either thrombin or APC [85]. This was a critical finding by these researchers, who further found that the compartmentalization of PAR1 bound to EPCR in cholesterol-enriched caveolar lipid rafts allowed it to act via pertussis-sensitive G proteins to assist in APC-mediated cytoprotection [86]. Alternatively, there is strong evidence that β-arrestins can mediate cytoprotective effects via PAR1 in endothelium (right pathway in Figure 4). Schuepbach [87] as well as Mosnier and coworkers [88] demonstrated that the anticoagulant activated protein C (APC) can cleave the N-terminus of PAR1 at an alternative site (Arg46), which is not observed with thrombin (cleavage site Arg41). The resulting signaling is biased towards β-arrestin, which was shown by Trejo and coworkers to be responsible for the cytoprotective effects of APC at PAR1 on endothelial cells [89,90].

## 3. PAR1, APC, and Neurodegeneration

**Figure 5:**
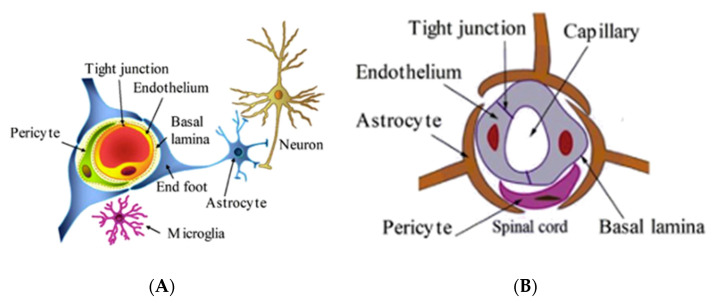
(**A**) Anatomy of BBB and (**B**) blood–spinal cord (BSCB) barrier.

**Figure 6:**
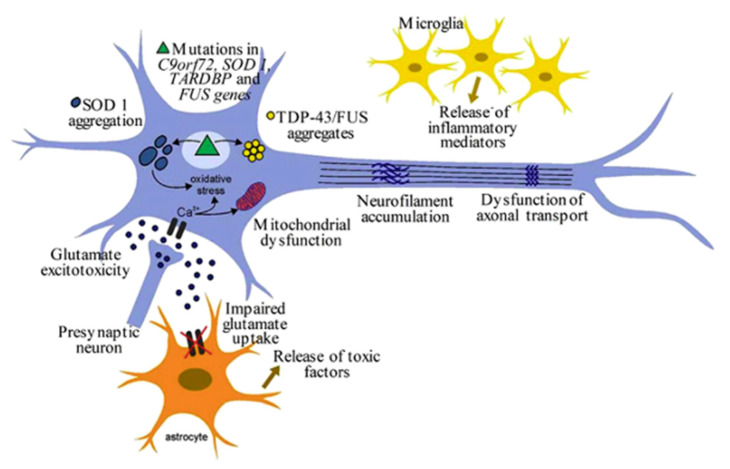
Overview of mutant SOD1 mouse model of ALS. Although emphasis has been placed on motor neuron degeneration, BBB dysfunction is also evident (as in Figure 5).

Why would APC be of value in neurodegenerative diseases such as ALS? In 2000, we showed that a naturally occurring, autosomal recessive mutant model of ALS, the wobbler mouse, overexpressed PAR1 prior to motor neuron degeneration [91]. In fact, motor neuron degeneration and death followed PAR1 overexpression both temporally and topographically in affected wobbler mice. Following publication of our observations with wobbler mice and PAR1, we developed data that showed that PAR1 mRNA was also upregulated in the spinal cords of SOD1 knockout mice (anatomical diagrams in Figure 5 and Figure 6).

### BBB Dysfunction in Neurodegenerative Diseases and APC as a Therapeutic

Numerous authors have published extensively on BBB dysfunction in AD [92,93,94,95,96]. In addition, a long history of BBB dysfunction in ALS patients had been previously reviewed [97]; however, in the “modern era”, Garbuzova-Davis and colleagues at the University of South Florida began to study BBB integrity in transgenic SOD1 mice in the early 2000s, finding evidence of BBB and blood–spinal cord barrier (BSCB) dysfunction both in this ALS model as well as in patients with ALS [98,99,100]. An overview of phenotypic neuronal changes in the SOD1 mouse model of ALS is given in Figure 6. Their work was the first to clearly show microvascular damage resulting from EC degeneration [100], and their work has continued over the last decade. In 2008, Zlokovic and colleagues at the University of Rochester reported microhemorrhages in the spinal cords of different transgenic SOD1 mouse models [101]. The implication held by us and others was that inhibition of thrombin-driven inflammatory PAR1 signaling could be beneficial in the context of neurodegeneration.

More recently, from studies of APC and its variants, it has become clear that certain ligands could block pro-inflammatory PAR1 signaling (e.g., driven by thrombin) while concurrently promoting anti-inflammatory or cytoprotective signaling via PAR1. APC has demonstrated the ability to indirectly deactivate thrombin and also bias PAR1, perhaps in complex with neighboring receptors, towards an anti-inflammatory and cytoprotective signaling pathway. In searching for a treatment for frequently fatal severe sepsis, researchers identified recombinant APC, which does not require TM activation, and developed this into the sepsis treatment drotrecogin alfa (activated), marketed as Xigris™ by Eli Lilly. It is an anticoagulating, anti-inflammatory, and profibrinolytic molecule, and some evidence has suggested that one mechanism for its efficacy was via EC cytoprotection related to the EPCR–APC-PAR1 signaling pathway. FDA approval came in the U.S. following the Phase 3 PROWESS clinical trial of 1690 patients in 2001 [102], although controversy followed post-approval. There was concern for bleeding events, although serious bleeding was only reported in 3.5% of patients in the PROWESS study. The European Medicines Agency required a confirmatory, placebo-controlled trial, PROWESS-SHOCK, which was published in 2012 and which showed no benefit [35]. Following this, Eli Lilly voluntarily withdrew Xigris™ from the market. A number of other articles were published, including a meta-analysis in 2012 [103], which found the potential for significant reduction in mortality with Xigris™ but a higher rate of severe bleeding compared to the original PROWESS trial. It seems apparent that dose-limiting bleeding effects give a therapeutic window for APC that is too narrow to be broadly useful in real-world situations of severe sepsis.

To address this challenge, Griffin, Zlokovic, and colleagues developed modified versions of APC that have greatly diminished anticoagulant activity but retain their cytoprotective activity, which is thought to be due to biased signaling via PAR1 activation [104,105]. One example, 3K3A-APC, appears to be neuroprotective in ischemic stroke and may be adjunctive in enhancing tissue plasminogen activator (tPA) safety for intra-arterial or fibrinolytic therapy. It has been successfully tested in a phase 2a trial for ischemic stroke [106,107]. These variants are clearly relevant to neuro-thromboinflammation in neurodegenerative diseases [108] and trauma, as described below.

In 2009, Zlokovic and colleagues reported that APC and non-anticoagulant APC analogs slowed disease progression and extended survival after disease onset in SOD1 ALS mice [109]. Although there has been some controversy over microhemorrhages in the SOD1 mice, using post-mortem tissue from ALS patients compared with non-neurodegenerative controls, attesting to the BBB, or more specifically blood–spinal cord barrier (BSCB) breakdown, they found perivascular deposits of erythrocyte-derived hemoglobin and hemosiderin, suggestive of erythrocyte extravasation [110]. Within spinal cord parenchyma they also found accumulation of plasma-derived immunoglobulin G, fibrin, and thrombin in ALS patients but not in controls [110]. These and other neurovascular and neuroinflammatory aspects of ALS were reviewed by Evans and colleagues [111]. Subsequently, in 2014, Zlokovic and colleagues showed that BSCB breakdown contributed to early motor neuron degeneration in ALS mice, and that restoring BSCB integrity via treatment with coagulation-deficient engineered variants of APC (5A-APC; RR230/231AA and KKK192-194AAA) early in the disease also slowed motor neuron degeneration [112].

In 2019 Zlokovic, then at the University of Southern California (USC) and collaborating with members of the Eli and Edythe Broad CIRM Center for Regenerative Medicine and Stem Cell Research at USC, reported in studies unrelated to BBB dysfunction that motor neurons induced either from ALS patients carrying the C9ORF72 hexanucleotide mutation or from several sporadic ALS patients’ iPSCs (iMNs) showed impaired autophagosome formation along with aberrant accumulation of glutamate receptors [113]. They stated that treatment of these iMNs with 3K3A-APC (i) reduced the amount of C9ORF72 dipeptide repeat protein (DPR), (ii) restored nuclear TDP-43 localization, and (iii) prolonged survival of both C9ORF72 and sporadic ALS iMNs [PMID: 31310593]. Furthermore, they showed that 3K3A-APC treatment also lowered glutamate receptor levels and rescued proteostasis in vivo in both C9ORF72 gain- and loss-of-function C9-BAC mice, as shown by Baloh and colleagues [114].

Based on these tissue culture and mouse model studies, ZZBiotech, founded by Zlokovic, is now funding a phase 2 clinical trial run by Dr. Dominic Rowe at Macquarie University Hospital in Sydney, Australia, to determine the safety and tolerability of 3K3A-APC in ALS patients. As stated on the website, they will be “looking at several biomarkers of disease activity” and a dose escalation regimen will be followed. The trial will also utilize PET imaging to determine whether microglial activation can be modulated over two weeks, as well as other biomarkers of microglia and monocyte activation.

## 4. Other PAR1 Ligands with Potential Neuroprotective Activity

**Figure 7:**
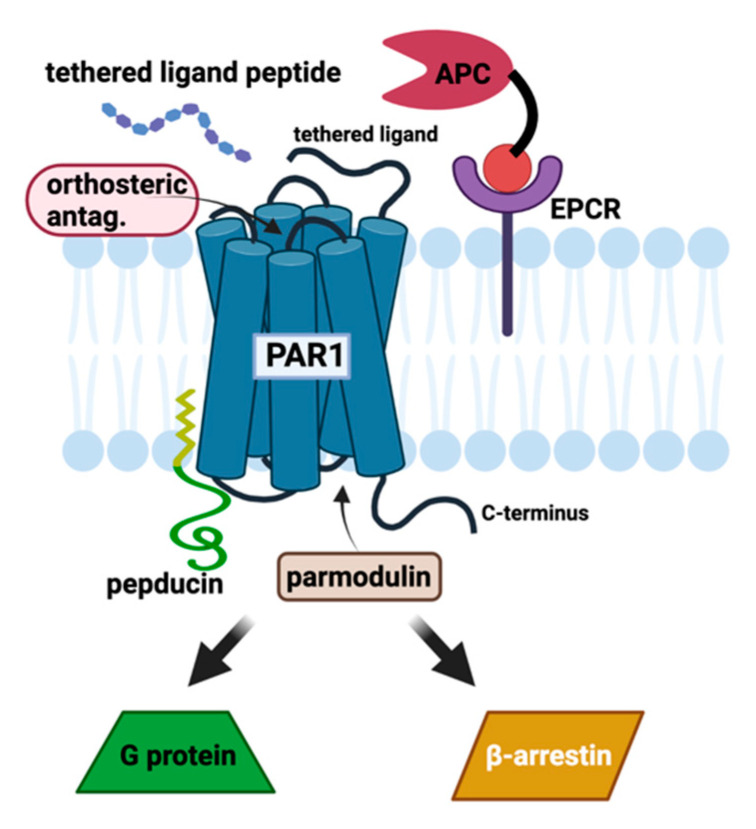
Modulators of PAR1 signaling. Recombinant wild-type and non-anticoagulant variants of APC drive cytoprotective PAR1 signaling in an EPCR-dependent fashion. This function is necessary for most of the described therapeutic activities of recombinant APC in preclinical animal models of acute and chronic inflammation. Cleavage of the N-terminus of PAR1 at Arg46 reveals a tethered ligand that leads to β-arrestin-mediated protective signaling. This effect can be mimicked with tethered ligand peptides such as TR47. Orthosteric PAR1 antagonists are competitive with the tethered ligand at the N-terminus of PAR1, and examples such as vorapaxar bind to the upper portion of PAR1 with a very low off-rate to block agonist-induced receptor activation and cell signaling. Pepducins are biomimetic peptides tethered to fatty acid membrane anchors that can modify PAR1 signaling and have been shown both to agonize and to antagonize PAR1. Parmodulins allosterically bind PAR1 at a putative intracellular site to modulate downstream PAR1 signaling outcomes, presumably through differential G protein and β-arrestin recruitment.

Besides APC and its variants, several classes of ligands of PAR1 and its interacting proteins have been developed for therapeutic purposes. These classes are summarized briefly below and in Figure 7.

### 4.1. Orthosteric PAR1 Antagonists and Inhibitors of PAR1/Thrombin Binding

We deem compounds that are likely to be competitive (in a mechanistic binding sense) with the PAR1 tethered ligands as orthosteric PAR1 antagonists. These compounds, represented by examples such as RWJ-58259 [115], atopaxar [76], and vorapaxar [116], are thought to bind near the top of PAR1, according to a vorapaxar-PAR1 X-ray structure [117]. A major disadvantage is that their binding tends to be poorly reversible, a property that is necessary to compete with the high effective concentration of the tethered ligand but which may introduce dangerous overdose and bleeding risks. Another disadvantage is that they may block all forms of PAR1 signaling, both pathological and beneficial. Flaumenhaft reported that vorapaxar induces apoptosis and barrier dysfunction at nanomolar concentrations in cultured endothelial cells [118].

Despite these potential complications, Shavit-Stein and colleagues recently reported on PAR1 as a therapeutic target in familial ALS with studies using G93A SOD1 transgenic mice [119]. For their studies, they centered their attention on comparing survival, weight, and motor analyses using rotarod scores with thrombin activity, PAR1 expression, and its localization in the cerebellum and cortex. They also incorporated a treatment trial into their two year study, in which they evaluated three different therapies directed at the PAR1 signaling pathway: (1) the thrombin inhibitor TLCK, (2) the PAR1 (presumably orthosteric) antagonist SCH 79797, and (3) FTS, an inhibitor of the Ras intracellular pathway. They found that each therapy targeting different components of the PAR1 cellular pathway extended survival, except for high-dose FTS. The greatest survival effect, a 10 day prolongation, was found with SCH 79797. In searching for molecular and pathologic confirmation, they found that brain thrombin activity was increased in SOD1 mouse brains in motor areas such as the brainstem, cerebellum, and the primary motor area in the posterior frontal cortex. Furthermore, in these same brain areas immunocytochemistry identified disruption of both PAR1 and GFAP astrocytic staining surrounding large pyramidal neurons.

In contrast to what we had found more than 20 years ago with wobbler mice, an autosomal recessive, naturally occurring ALS model [91], PAR1 levels were reduced in SOD1 Tg mice [119]. Our data indicated that in phenotypic degenerating wr/wr spinal cords where motor neurons were progressively being lost, wr/wr astrocytes produced higher α-thrombin activity levels and a PAR1-specific antibody showed markedly increased protein levels on motor neurons in the cervical cord ventral horn than in wild-type littermates [91].

Shavit-Stein and colleagues recently produced an α-thrombin/PAR1 inhibitor based on the N-terminal thrombin binding site of PAR1, which spares the anticoagulation activity of thrombin and is composed of five amino acids within the PAR1 tethered ligand sequence, named PARIN5 [120]. They designed PARIN5 based on the thrombin-specific recognition site sequence (35NATLDPR41) with a 5-amino acid backbone and reported on its efficacy in a mouse model of diabetic neuropathy. Although this molecule may block undesirable thrombin-driven activation of PAR1, it also would presumably not drive any beneficial anti-inflammatory or cytoprotective PAR1 signaling on its own.

### 4.2. Anti-Inflammatory PAR1 N-Terminal Peptides

Schuepbach [87] and Mosnier [88] separately reported that peptides derived from the cleavage of the N-terminus of PAR1 by APC at Arg46 demonstrated endothelial barrier-protective and cytoprotective effects in a manner mimicking APC. It is unclear whether these peptides (an example is termed TR47 by Mosnier) have sufficient potency and stability for therapeutic applications, although they may represent promising lead compounds in anti-inflammatory drug discovery.

### 4.3. Pepducins

Another category of PAR1-targeted ligands is biomimetic lipid-tethered peptides that mimic the intracellular loops of GPCRs and can allosterically inhibit or promote GPCR engagement with G proteins. These pepducins, developed by Kuliopulos, Covic, and coworkers, have been developed for several GPCRs [121,122,123]. Regarding PAR1, they have been proposed primarily for use as cancer [124] and cardiovascular therapeutics, and the pepducin PZ-128 has reached clinical stages for coronary artery disease [125]. The disadvantages of pepducins include the need for i.v. dosing and uncertain tissue and BBB permeability.

### 4.4. Parmodulins

**Figure 8:**
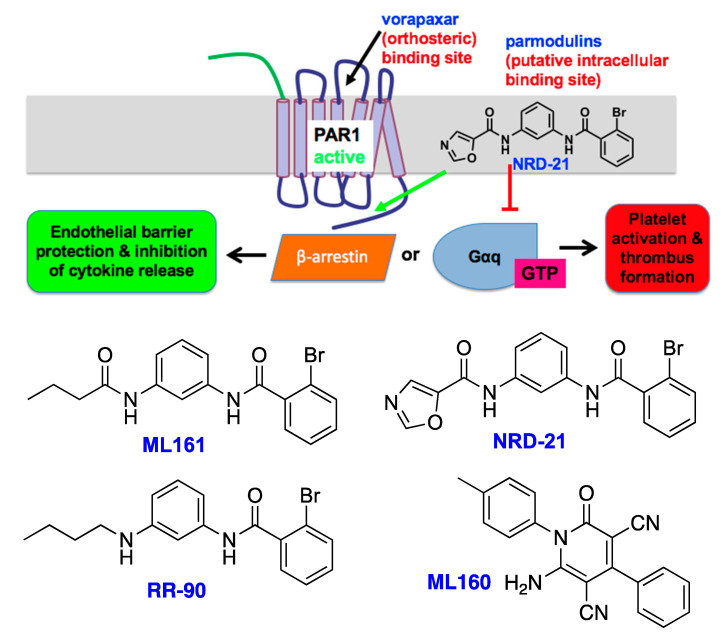
Top: Simplified parmodulin mode-of-action diagram. Inflammatory signaling (typically driven by α-thrombin) at PAR1 can be blocked by parmodulins, which bind to a putative intracellular site. Anti-inflammatory signaling of PAR1, thought to be mediated by β-arrestin 2, may be permitted or promoted by parmodulins. Bottom: Several key parmodulins discovered in the Dockendorff lab.

Flaumenhaft and colleagues discovered the first example of a small-molecule allosteric modulator of PAR1 [126], and in collaboration with the Dockendorff lab, further examples have been discovered and pharmacologically profiled. These compounds, termed parmodulins, possess an unusual putative intracellular binding site, thought to be near helix 8 at the cytoplasmic face of PAR1, as indicated by modeling studies [126] and calcium mobilization assays with chimeric receptors [118]. The initial lead parmodulin, ML161 [127], possesses both antithrombotic and anti-inflammatory activities. Parmodulins can inhibit the PAR1-driven activation of platelets and endothelial cells at nanomolar concentrations, although unlike typical orthosteric antagonists, this activity is reversible. They appear to act as biased ligands of PAR1, inhibiting certain functions (e.g., Gq-driven calcium mobilization), but not others (G12/13-driven shape change in platelets). They have also demonstrated in vitro and in vivo activities in various models of inflammation, including TNF-α-driven endothelial TF expression [128] and inhibition of arterial thromboinflammation in laser-injured, LPS-dosed mice [129]. Isermann also reported that ML161 mimics the action of APC and is highly tissue-protective in a mouse model of coronary ischemia–reperfusion injury [130], and very recently reported that ML161 (also known as parmodulin 2) returned kidney function to near-baseline levels in a mouse model of diabetic nephropathy [131]. Several parmodulins reported by the Dockendorff lab are shown in Figure 8.

## 5. Additional Rationale for PAR1 as a Therapeutic Target for ALS

It is an understatement, of course, that effective therapies for ALS treatment and prevention are urgently needed. It has been a guiding principle of the author’s (B.F.) translational research with ALS over many decades to focus on thromboinflammation, before this was even a useful term. As reviewed above, at the crux of thromboinflammation is the small family of GPCRs known as PARs, with PAR1 being the best known example and the one first targeted for this “war to the knife, and knife to the hilt.” One objective in the authors’ labs is to confirm that anti-inflammatory small-molecule modulators of PAR1 (the parmodulins) possess neuroprotective effects and acceptable drug-like properties (including safety and selectivity) that will justify their preclinical development to ultimately treat and potentially prevent ALS and other neurodegenerative diseases. Given the BBB dysfunction that is confirmed for ALS, targeting PAR1 on BMECs may have additional significant therapeutic value, for example in TBI, given thrombin’s role in increasing barrier permeability by activating BBB/BMEC PAR1 [132]. In the process of arriving at this goal, we will also identify biomarker(s) relevant to the mode of action of these compounds that correlate with efficacy in mouse model(s) of ALS, such as the C9orf72 [133]. Such biomarkers might be in the form of purified exosomes carrying cargoes that include neuronal, endothelial, and innate immune cell molecules. In so doing, we hope to produce a PAR1 ALS theranostic. What are the factors, beyond what we have reviewed already, that have led us to this conclusion?

Thromboinflammation is correlated with ALS and other neurodegenerative disorders. It has become increasingly clear that following systemic infection or tissue injury, a coordinated activation of inflammatory (innate immune) and hemostatic (coagulation) responses occurs. Although a number of years ago it was proposed that similar synchronized responses lie behind the development of neurodegenerative diseases such as ALS, only recently has experimental evidence for this hypothesis accumulated significantly. Other than genetic causes, the most common antecedent event for ALS and other neurodegenerative diseases is head injury or traumatic brain injury (TBI).

PAR1 drives important pro- and anti-inflammatory signals in different cell types, which are now leveraged in the clinic. Again, a key player in both coagulation and inflammation is PAR1, which acts as the receptor for thrombin, one of a small family of GPCRs that “self-activate” by cleavage of their N-termini by various proteases.

APC has demonstrated neuroprotective effects in models of ALS. As we describe above, since 2009, Zlokovic and colleagues have provided evidence that APC or APC analogs lacking anticoagulant activity slowed disease progression and extended survival of SOD1 mice with ALS-like characteristics, an effect that required PAR1 and PAR3 and that led to decreased expression of mutant SOD1 [109]. More recently, 3K3A-APC was found to increase survival and rescue several key defects in induced motor neurons derived from stem cells from both familial and sporadic ALS patients, including proper TDP-43 localization and accumulation of glutamate receptors [113]. Of special note is that 3K3A-APC is presently in a phase 2 trial for ALS in Sydney, Australia.

Furthermore, 3K3A-APC has disadvantages that may complicate chronic administration. Although the biology and molecular biology underlying the utility of APC at the PAR1 target for ALS is strong, the uncontrolled bleeding complication of wild-type APC is unacceptable, and so the development of anticoagulation-deficient variants such as 3K3A-APC have apparently eliminated this issue; however, 3K3A-APC is a protein with a molecular mass of 62 kDa, essentially the same as wild-type PC. It also requires i.v. administration and has a short plasma half-life (<0.5 h) [134]; therefore, at least in its present form, it is unclear whether longer term chronic dosing is feasible. Furthermore, although movement across the BBB/NVU (neurovascular unit) is proposed to occur by binding to EPCR and transcytosis [135], this was preclinical in mice and is not yet confirmed in humans. In addition, although preclinical studies indicated that 3K3A-APC was roughly 80% less active than wild-type APC as an anticoagulant, frequent clinical clotting assays such as partial thromboplastin time and prothrombin time assays may need to be performed. Consequently, an orally active, non-anticoagulant, small-molecule therapy for ALS based on the PAR1 target would be highly desirable, and relative to APC could also offer improvements in half-life and brain penetration. Parmodulins likely represent the best current lead compounds in this regard (see Conflicts of Interest section).

## 6. Concluding Remarks

The nexus of coagulation and inflammation has emerged as an important but still underexplored region for drug discovery, particularly with regards to neurodegenerative diseases for which new approaches have been lacking and new therapies are desperately needed. Biomarkers that indicate elevated levels of neuro-thromboinflammation, and correlation of these biomarkers to disease progression, may be needed to translate relevant animal models to the clinic. Validated biomarkers will also help to justify the therapeutic use of suitable antithrombotic or anti-inflammatory compounds, such as those able to enhance the integrity of damaged blood–CNS barriers.

A potential clue to the pathogenesis of neurodegenerative diseases such as ALS may be gleaned from reviewing adverse events arising from the phase III vorapaxar clinical trials. Surprisingly, there was an increased number of ALS diagnoses in the treatment arms compared to the placebo arms of the TRACER [78] and TRA2P [136] phase 3 trials; however, the low number of events (2 vs. 4 diagnoses in ~35,000 patient-years of exposure in each pooled arm of the two studies) is unlikely to have reached statistical significance. This was not mentioned in the publications of the results but was discussed in the FDA’s analysis of the vorapaxar NDA data [137,138]. Data beyond those obtained in the maximum 3 year trial would obviously be desirable. This possible vorapaxar–ALS association may fit with previous studies carried out by us and others on neuro-thromboinflammation over the past several decades. As mentioned, vorapaxar inhibits all signaling downstream of PAR1, and perhaps the resulting lack of beneficial PAR1 signaling at the BBB or in the nervous system increases the relatively rare risk of ALS, suggesting that therapies that increase beneficial PAR1 signaling could be useful for the prevention or treatment of ALS and other neurodegenerative diseases. Successful clinical trials with 3K3A-APC or other PAR1 ligands will ultimately support this hypothesis, although the modulation of other targets downstream from PAR1 may also prove to be valuable.
